# Retraction: Schwannoma: A Rare Etiology of Pancoast Syndrome

**DOI:** 10.7759/cureus.r94

**Published:** 2024-01-25

**Authors:** Fahad A Alshammari, Abdulmohsen M Alotaibi, Mahdi A Alali, Nawaf S Alkhileiwi, Sultan M Alshammari, Mansour T Albagami, Yasser G Alarimah, Faisal A Aldughaim, Kawther A Alsadady, Faisal F Alshammari, Kouther M Alhedires, Noor A Albejais, Mohammed F Alharbi, Awadh M Alharthi, Malak Alshammari

**Affiliations:** 1 Family Medicine, Imam Abdulrahman Bin Faisal University, Dammam, SAU; 2 Family Medicine, King Abdulaziz Hospital, Taif, SAU; 3 Family Medicine, King Faisal University, Hofuf, SAU; 4 Family Medicine, Umm Al-Qura University, Mecca, SAU; 5 Family Medicine, AlMaarefa University, Ad Diriyah, SAU; 6 Family Medicine, Dar Al Uloom University, Riyadh, SAU; 7 Family Medicine, University of Hail, Hail, SAU; 8 Family Medicine, Prince Saud Bin Jalawy Hospital, Al-Mubarraz, SAU; 9 Family Medicine, Qassim University, Qassim, SAU; 10 Family Medicine, King Faisal Hospital, Taif, SAU; 11 Internal Medicine, Imam Abdulrahman Bin Faisal University, Dammam, SAU

The Editors-in-Chief have retracted this article. Concerns were raised regarding the identity of the authors on this article. Specifically, Faisal Alhaway and Malak Shammari have stated that they were added to this article without their knowledge or approval. The identity of the other authors could also not be verified. As the appropriate authorship of this work cannot be established, the Editors-in-Chief no longer have confidence in the results and conclusions of this article.

